# Association of Sleep Disturbance With Survival After Colorectal Cancer Diagnosis: Results From the ColoCare Study

**DOI:** 10.1002/cam4.71576

**Published:** 2026-01-26

**Authors:** Anita R. Peoples, Victoria Damerell, Jennifer Ose, Erin M. Siegel, Tengda Lin, Sheetal Hardikar, Caroline Himbert, Mmadili N. Ilozumba, Petra Schrotz‐King, Sylvia L. Crowder, Adetunji T. Toriola, David Shibata, Christopher I. Li, Doratha A. Byrd, Elena S. Aßmann, Heather S. L. Jim, Jane C. Figueiredo, Cornelia M. Ulrich, Biljana Gigic

**Affiliations:** ^1^ Huntsman Cancer Institute Salt Lake City Utah USA; ^2^ Department of Population Health Sciences University of Utah Salt Lake City Utah USA; ^3^ American Cancer Society Atlanta Georgia USA; ^4^ Department of General, Visceral, and Transplantation Surgery Heidelberg University Hospital Heidelberg Germany; ^5^ Department of Media, Information, and Design University of Applied Sciences and Arts Hannover Germany; ^6^ Division of Population Science, Department of Cancer Epidemiology H. Lee Moffitt Cancer Center and Research Institute Tampa Florida USA; ^7^ Department of Epidemiology Harvard T. H. Chan School of Public Health Boston Massachusetts USA; ^8^ National Center for Tumor Diseases (NCT) NCT Heidelberg, A Partnership Between DKFZ and University Hospital Heidelberg Heidelberg Germany; ^9^ Division of Primary Cancer Prevention German Cancer Research Center (DKFZ) Heidelberg Heidelberg Germany; ^10^ Department of Health Outcomes and Behavior H. Lee Moffitt Cancer Center and Research Institute Tampa Florida USA; ^11^ Washington University School of Medicine in St. Louis St. Louis Missouri USA; ^12^ University of Tennessee Health Science Center Memphis Tennessee USA; ^13^ Division of Public Health Sciences Fred Hutchinson Cancer Center Seattle Washington USA; ^14^ School of Medicine Technical University of Munich Munich Germany; ^15^ Department of Medicine Samuel Oschin Comprehensive Cancer Institute, Cedars‐Sinai Medical Center Los Angeles California USA

**Keywords:** colorectal cancer, disease‐free survival, overall survival, recurrence, sleep disturbance, survivorship

## Abstract

**Introduction:**

Sleep problems are common among cancer patients. The relationship between sleep disruption and clinical outcomes after colorectal cancer (CRC) diagnosis remains poorly understood. We investigated associations of sleep disruption with survival and recurrence in patients with CRC.

**Methods:**

CRC patients with stages I–IV (*N* = 895) were included in this study. Self‐reported sleep disturbance was assessed presurgery using the sleep item from the European Organization for Research and Treatment of Cancer Quality of Life Questionnaire‐Core‐30 and classified into “no/mild” or “moderate/severe” sleep disturbance. Cox‐proportional hazard models were computed (HRs and 95% confidence intervals) to investigate associations of sleep disturbance with overall survival (OS), disease‐free survival (DFS), and risk of recurrence, adjusting for age, sex, body mass index, tumor stage and site, and study site.

**Results:**

Thirty percent of patients reported moderate/severe sleep disturbance. *N* = 190 (21%) were deceased after a median follow‐up of 31 months, whereas 74 patients (15%) had a recurrence. Patients with moderate/severe vs. no/mild sleep disturbance had worse OS (HR = 1.46; 95% CI = 1.07–1.98; *p* = 0.02). There were no significant associations for sleep disturbance with DFS and risk of recurrence. Stratified analyses indicated that the worse OS rates due to sleep disturbance were stronger in patients who were middle‐aged and older, male, overweight/obese, diagnosed with rectal cancer and stage I–III.

**Conclusions:**

Poor sleep is common among CRC patients and is associated with worse overall survival. These findings highlight the potential value of preoperative sleep screening as a way to identify patients at higher risk of poor outcomes, warranting further investigation in future studies.

**Trial Registration:**

ClinicalTrials.gov identifier: NCT02328677

## 1 Introduction

Colorectal cancer ranks as the second leading cause of cancer‐related mortality, with 1.6 million deaths per year estimated for 2040 [1, 2]. Although clinical and pathologic characteristics, including tumor stage and treatment, are some of the main predictors of colorectal cancer prognosis [3, 4], limited emerging research highlights the importance of behavioral and lifestyle risk factors in influencing clinical outcomes such as survival and recurrence among patients with colorectal cancer [5, 6]. Thus, it is critical to identify modifiable risk factors of colorectal cancer outcomes with the potential to develop tailored mitigation strategies for improving prognosis.

Lifestyle factors such as poor sleep have been shown to be associated with the increased risk of many cancers [7, 8], including colorectal cancer [9]. Poor sleep is also highly common among cancer patients and survivors and includes problems such as sleep disturbance, poor sleep quality, sleep fragmentation, insufficient sleep duration, daytime sleepiness, and/or insomnia [10]. Current longitudinal studies suggest that up to 87% of newly diagnosed or recently treated cancer patients report poor sleep [10, 11], with one recent study reporting that 70% of colorectal cancer patients had trouble sleeping [12], underscoring the need to assess sleep as a potential risk stratification tool in survivorship care.

In recent years, the role of sleep in cancer progression and survival has received increasing attention. Several studies have indicated that poor sleep may be linked to reduced survival and higher recurrence rates in cancer patients [13, 14, 15, 16, 17]. However, only a few studies have specifically evaluated the association between self‐reported sleep and survival or recurrence in colorectal cancer populations, with mixed findings [18, 19]. For instance, a meta‐analysis of 32 studies across various cancer types reported that both short (< 6 h) and long (> 9 h) sleep durations were associated with increased all‐cancer‐specific mortality, suggesting a U‐shaped relationship, although no statistically significant associations were observed between sleep duration and colorectal cancer‐specific mortality [13]. Similarly, a long‐term follow‐up study using data from the Nurses' Health Study found that longer sleep duration (≥ 9 h) and sleep difficulties were associated with increased all‐cause mortality in women with breast cancer [14]. In contrast, another study among early‐stage breast cancer survivors reported that consistent short (≤ 6 h) or long (≥ 9 h) sleep duration were not risk factors for poor prognosis [16]. Among colorectal cancer‐specific studies, one prospective cohort found that short sleep duration (< 5 h) was associated with a 36% increased risk of all‐cause mortality and a 54% increased risk in colorectal cancer‐specific mortality [19]. However, another study did not observe significant associations between short (≤ 6 h) or long (≥ 9 h) sleep duration and all‐cause mortality [20]. Although much of the prior research has focused on sleep duration, relatively few studies have examined other dimensions of sleep, such as sleep disturbance. To date, only one study has evaluated subjective sleep disturbance before and during chemotherapy and found that sleep was an independent prognostic factor of overall survival; however, this study was limited to patients with metastatic colorectal cancer [18]. Thus, these findings underscore the need to better understand the relationship between different dimensions of sleep and survival among colorectal cancer survivors, especially those with stage I–III disease, in whom long‐term prognosis may be more modifiable.

The primary aim of this study was to assess the association between sleep disturbance and overall survival after a diagnosis of stage I–IV colorectal cancer. We also examined the association of sleep disturbance with secondary outcomes of disease‐free survival and risk of recurrence. We hypothesized a priori that preoperative sleep disturbance may serve as a prognostic indicator of overall survival in colorectal cancer patients.

## 2 Methods

### 2.1 Study Design and Participation Selection

The ColoCare Study is a large, international, multicenter prospective cohort of newly diagnosed stage I–IV colorectal cancer patients [21]. The design of the ColoCare Study has previously been described [21, 22, 23, 24]. Briefly, the ColoCare Study aims to investigate the etiology, prognosis, and outcomes in colorectal cancer survivorship. The eligibility criteria include newly diagnosed colon (ICD‐10 C18), rectum, or rectosigmoid cancer (ICD‐10 C19/C20); adenocarcinoma; 18 years of age or older at the time of recruitment; histopathologically confirmed invasive cancer according to the American Joint Committee on Cancer (AJCC) classification system, that is, stage I–IV; and ability to provide informed consent. Patients who meet the inclusion criteria are recruited at the time of primary surgery (baseline), and followed via questionnaire administration and medical chart abstractions up to 60 months posttreatment. Baseline self‐reported questionnaires administered at study enrollment include demographic and clinical information, medical history, anthropometric measurements, lifestyle factors and health behaviors, patient‐reported symptoms, and health‐related quality of life.

In this study, we included patients diagnosed with stage I–IV colorectal cancer, enrolled between December 2009 and April 2021 at the ColoCare Study sites in the U.S. at Moffitt Cancer Center (MCC) and in Germany at the National Center for Tumor Diseases (NCT) in Heidelberg and the University Hospital (UKHD) Heidelberg, who underwent surgery, did not die due to postsurgical complications (i.e., death within 30 days after surgery), and had available data on sleep disturbance and covariates at baseline. The study was approved by the institutional review board of the Moffitt Cancer Center and by the Ethics Committee of the University of Heidelberg, and all participants provided written informed consent.

### 2.2 Measures

#### 2.2.1 Demographic and Clinical Characteristics

Baseline questionnaires were used to assess demographic (age at diagnosis, sex, race/ethnicity) and behavioral (smoking, BMI) characteristics. Clinical characteristics including stage at diagnosis and primary tumor site, as well as adjuvant and neoadjuvant treatment, were abstracted from medical records.

#### 2.2.2 Sleep Disturbance Exposure Assessment

Subjective sleep disturbance at baseline was assessed using the corresponding scale of a validated, multidimensional questionnaire of health‐related quality of life, namely the European Organization for Research and Treatment of Cancer Quality of Life Questionnaire (EORTC QLQ‐C30), which includes “[During the past week] Have you had trouble sleeping?”, with four answer choices (not at all, a little, quite a bit, very much) [25]. The EORTC QLQ‐C30 sleep scale has been extensively used in other cancer cohorts and has been validated as indicated by a correlation of 0.73 with the Jenkins Sleep Scale assessed in a large sample of mixed cancer patients [26], as well as correlations ranging from 0.74–0.81 with the Jenkins Sleep Scale assessed in colorectal cancer patients [27]. Another study in esophageal cancer patients also showed strong associations between the EORTC QLQ‐C30 sleep scale and the 18‐item Karolinska Sleep Questionnaire (odds ratio of 8.2) [28]. A sleep score from EORTC QLQ‐C30 sleep scale was calculated as described in the EORTC QLQ‐C30 scoring manual [29]. This process involved transforming the raw severity scores into a linear scale ranging from 0 to 100. A higher score represents greater sleep disturbance. Although no established cutoff scores currently exist for the EORTC QLQ‐C30 sleep scale, we categorized participants who reported “not at all” or “a little” trouble sleeping as having good sleep and those who reported “quite a bit” or “very much” trouble sleeping as having poor sleep. The transformation of the sleep item was retained for reporting consistency [30, 31], although it was not necessary for the dichotomized analysis of sleep disturbance in the final models.

#### 2.2.3 Survival Outcome and Risk of Recurrence

For detailed information on clinical outcomes, standardized medical chart abstractions and linkages to cancer registry and vital status records were performed. In particular, information on surgical procedures was obtained through medical record abstractions at least 2 years after diagnosis. Survival was determined through patients' internal and external medical records, routine follow‐up mailings, state or national cancer and death registries. Recurrence was determined via medical records, including imaging and pathology reports postsurgery. All‐cause mortality was defined as the time from surgery to the date of death (irrespective of the cause) or last contact, whichever occurred first. Recurrence was defined as new recurrence of colorectal cancer after complete surgical removal of the tumor, based on resection status. For the analysis of disease‐free survival, we excluded patients who were diagnosed with stage IV disease, recurrence within 30 days postsurgery, or noncurative resections (R1, R2, or Rx), since these patients were not considered disease free postsurgery. Disease‐free survival time was defined as the time from surgery to either date of disease recurrence, death, or last contact, whichever occurred first.

### 2.3 Statistical Analyses

The main outcome was overall survival, as defined as the time from study enrollment to death from any cause or the last follow‐up. Secondary outcomes were disease‐free survival and risk of recurrence. Means and standard deviations were calculated for continuous variables, while percentages were used to describe categorical variables. Cox proportional hazard regression models were used to estimate the hazard ratios (HR) and 95% confidence intervals (95% CI) for the associations of sleep disturbance with overall survival, disease‐free survival, and risk of recurrence. Models were adjusted for potential confounders and covariates, namely age at diagnosis, sex, BMI, tumor site and stage, and study site, which were selected a priori based on existing literature. The proportional hazards assumptions were tested with *χ* ^2^‐test and Schoenfeld residuals. The final sample size for risk of recurrence and disease‐free survival analysis was *n* = 482.

For the main outcome of overall survival, we further conducted stratified analyses by age (< 50 years or ≥ 50 years), sex (male or female), BMI (normal weight or overweight/obese according to the World Health Organization (WHO) cutoffs), tumor site (colon or rectum) to identify effect modification. While surgery in stage I–III disease is typically performed with curative intent, patients with stage IV disease were also included if they underwent planned surgery for the primary tumor and/or metastases. We acknowledge that surgical intent in metastatic cases may vary (curative vs. palliative), and we therefore conducted stratified analyses by cancer stage (I–III vs. IV) to account for potential clinical heterogeneity. In each stratified analysis, the variable used for stratification was excluded from the model. We also added interaction terms to nonstratified models to identify whether age, sex, BMI, tumor site, or tumor stage modified the associations between sleep disturbance and overall survival. The significance of the interaction term was examined using likelihood ratio tests, which compared the model with and without the interaction terms. A two‐sided *p*‐value of < 0.05 was considered statistically significant for all tests. Statistical analyses were conducted using SPSS version 29 (IBM Corp., Armonk, NY). Overall survival curves stratified by sleep disturbance severity (no/mild vs. moderate/severe) were generated using the Kaplan–Meier method, and differences between groups were assessed with the log‐rank test.

## 3 Results

### 3.1 Study Population Characteristics

A total of 895 colorectal cancer patients were included in the analysis, with 30% of patients self‐reported moderate to severe levels of sleep disturbance at baseline (Table 1). The mean age was 62 years, 39% were female, the majority were White (96%), and about 70% were overweight or obese. Over half of the patients were diagnosed with rectal cancer (53%) and about 84% were diagnosed with stage I–III colorectal cancer.

**TABLE 1:** Characteristics of the study population.

Characteristics	Colorectal cancer patients (*N* = 895)
Age (years), mean (SD)	62.4 (±12.0)
Sex, *n* (%)	
Female	348 (38.9%)
Male	547 (61.1%)
Race, *n* (%)^a^	
White	858 (96.0%)
Non‐White	36 (4.0%)
BMI (kg/m^2^), mean (SD)	28.0 (±5.9)
Tumor stage, *n* (%)	
I	169 (18.9%)
II	259 (28.9%)
III	319 (35.6%)
IV	148 (16.5%)
Tumor site, *n* (%)	
Colon	425 (47.5%)
Rectum	470 (52.5%)
Neoadjuvant treatment, *n* (%)^a^	
No	444 (58.6%)
Yes	314 (41.4%)
Adjuvant treatment, *n* (%)^a^	
No	353 (47.2%)
Yes	395 (52.8%)
Resection status, *n* (%)^a^	
R0	639 (90.4%)
R1	36 (5.1%)
R2	7 (1.0%)
Rx	25 (3.5%)
Smoking status, *n* (%)^a^	
Never	399 (47.5%)
Ever	441 (52.5%)
Sleep disturbance, *n* (%)	
No/mild sleep disturbance	631 (70.5%)
Moderate/severe sleep disturbance	264 (29.5%)
Vital status	
Alive, *n* (%)	705 (78.8%)
Follow‐up time (month), median (SD)	32.6 (±29.6)
Deceased, *n* (%)	190 (21.2%)
Follow‐up time (month), median (SD)	24.5 (±24.1)
Recurrence^b^	
No, *n* (%)	408 (84.6%)
Follow‐up time (month), median (SD)	37.1 (±26.0)
Yes, *n* (%)	74 (15.4%)
Follow‐up time (month), median (SD)	15.7 (±14.3)

*Note:* Data might not add to 100% because of rounding.

Abbreviations: BMI, body mass index (kg/m^2^); SD, standard deviation.

^a^ Missing values due to nonresponse not shown [neoadjuvant treatment: *n* = 137 (15.3%); adjuvant treatment: *n* = 147 (16.4%); resection status: *n* = 188 (21.0%); smoking status: *n* = 55 (6.1%)].

^b^ Population at risk of recurrence is *n* = 482.

### 3.2 Association Between Sleep Disturbance and Overall Survival

**FIGURE 1:**
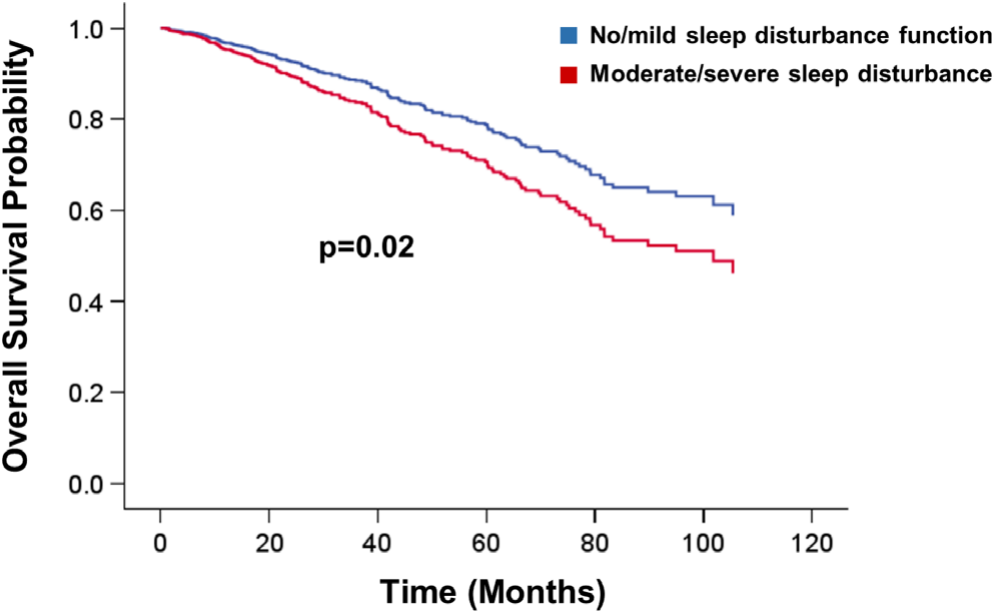
Associations between sleep disturbance and overall survival in colorectal cancer patients.

A total of 190 deaths (129 males, 61 females) occurred during follow‐up. Median follow‐up time was 33 months among those patients who were still alive at the end of the follow‐up period, while it was 31 months among all participants (Table 2 and Figure 1). The median overall survival time in patients with moderate to severe sleep complaints was 27 months as compared to 32 months among those reporting good sleep. The presence of moderate to severe sleep complaints at baseline was associated with a 47% higher risk of death (HR = 1.47; 95% CI = 1.09–1.98; *p* = 0.01) in unadjusted analysis. This association remained essentially unchanged after adjusting for study site, age, sex, BMI, tumor site, and stage at diagnosis with an estimated 46% increased risk of death (HR = 1.46; 95% CI = 1.07–1.98; *p* = 0.02).

**TABLE 2:** Adjusted cox proportional hazard models for the association between sleep disturbance and survival and risk of recurrence in colorectal cancer patients.

	Sleep disturbance	Total *N* (deaths)	Unadjusted		Adjusted	
			HR (95% CI)	*p*	HR (95% CI)	*p*
Overall survival	No/mild sleep disturbance	631 (124)	1.00 (Ref)		1.00 (Ref)	
	Moderate/severe sleep disturbance	264 (66)	1.47 (1.09–1.98)	**0.01**	1.46 (1.07–1.98)	**0.02**
Disease‐free survival	No/mild sleep disturbance	345 (72)	1.00 (Ref)		1.00 (Ref)	
	Moderate/severe sleep disturbance	137 (30)	1.17 (0.76–1.80)	0.47	1.19 (0.76–1.85)	0.45
Risk of recurrence	No/mild sleep disturbance	345 (51)	1.00 (Ref)		1.00 (Ref)	
	Moderate/severe sleep disturbance	137 (23)	1.21 (0.74–1.99)	0.44	1.12 (0.67–1.86)	0.68

*Note:* All analyses were adjusted for study site, age, sex, body mass index (kg/m^2^), tumor site, and stage at diagnosis. Statistically significant *p*‐values are highlighted in bold.

Abbreviations: 95% CI, 95% confidence interval; HR, hazard ratio.

The association between poor sleep and overall survival was significantly modified by age (*p*‐interaction = 0.02) such that poor sleep significantly increased the risk of death among patients 50 years and older (HR = 1.58; 95% CI = 1.15–2.19; *p* = 0.005, Table 3). The association between poor sleep and overall survival was also significantly modified by sex (*p*‐interaction = 0.01). A stronger association between poor sleep and worse overall survival was observed among male patients (HR = 1.57; 95% CI = 1.08–2.29; *p* = 0.02) compared to female patients. Additionally, stronger significant associations between poor sleep and worse overall survival were observed among overweight or obese patients (HR = 1.71; 95% CI = 1.18–2.49; *p* = 0.005), patients with rectal cancer (HR = 1.60; 95% CI = 1.03–2.47; *p* = 0.04), and patients with stages I–III colorectal cancer (HR = 1.64; 95% CI = 1.09–2.47; *p* = 0.02).

**TABLE 3:** Adjusted cox proportional hazard models for the association between sleep disturbance and overall survival in colorectal cancer patients, stratified by age, sex, body mass index, tumor site, and stage at diagnosis.

Sleep disturbance	Total *N* (deaths)	HR (95% CI)	*p*_trend_	Total *N* (deaths)	HR (95% CI)	*p*_trend_	*p*_interaction_
Age	< 50 years			≥ 50 years			
No/mild sleep disturbance	86 (13)	1.00 (Ref)		545 (111)	1.00 (Ref)		
Moderate/severe sleep disturbance	39 (4)	0.66 (0.20–2.23)	0.51	225 (62)	1.58 (1.15–2.19)	**0.005**	**0.015**
Sex	Female			Male			
No/mild sleep disturbance	217 (35)	1.00 (Ref)		414 (89)	1.00 (Ref)		
Moderate/severe sleep disturbance	131 (26)	1.23 (0.72–2.11)	0.45	133 (40)	1.57 (1.08–2.29)	**0.02**	**0.01**
BMI	Normal weight (< 25 kg/m^2^)			Overweight or obese (≥ 25 kg/m^2^)			
No/mild sleep disturbance	117 (39)	1.00 (Ref)		454 (85)	1.00 (Ref)		
Moderate/severe sleep disturbance	90 (22)	0.96 (0.56–1.66)	0.89	174 (44)	1.71 (1.18–2.49)	**0.005**	0.71
Tumor site	Colon			Rectum			
No/mild sleep disturbance	301 (67)	1.00 (Ref)		330 (57)	1.00 (Ref)		
Moderate/severe sleep disturbance	124 (31)	1.33 (0.86–2.08)	0.21	140 (35)	1.60 (1.03–2.47)	**0.035**	0.97
Tumor stage	Stage I–III			Stage IV			
No/mild sleep disturbance	534 (75)	1.00 (Ref)		97 (49)	1.00 (Ref)		
Moderate/severe sleep disturbance	213 (37)	1.64 (1.09–2.47)	**0.017**	51 (29)	1.30 (0.81–2.11)	0.28	0.91

*Note:* Analyses were adjusted for study site, age, sex, body mass index, tumor site, and stage at diagnosis accordingly. In each stratified analysis, the variable used for stratification was excluded from the model. Statistically significant *p*‐values are highlighted in bold.

Abbreviations: 95% CI, 95% confidence interval; BMI, body mass index (kg/m^2^); HR, hazard ratio.

### 3.3 Association of Sleep Disturbance With Disease‐Free Survival and Risk of Recurrence

Since patients who were diagnosed with stage IV cancers and patients who had a recurrence within 30 days postsurgery were excluded, the sample size for risk of recurrence or disease‐free survival analysis was *n* = 482. A total of 102 patients had either recurrence or were deceased after a median follow‐up time of 36 months. Out of these, only 15% of patients (*n* = 74) had recurrence with a median follow‐up time of 33 months. Poor sleep at baseline was not significantly associated with disease‐free survival in the adjusted model (HR = 1.19; 95% CI = 0.76–1.85; *p* = 0.45) analysis. Similarly, poor sleep was not significantly associated with risk of recurrence in the adjusted analysis (HR = 1.12; 95% CI = 0.67–1.86; *p* = 0.68).

## 4 Discussion

In this prospective cohort of stage I–IV colorectal cancer patients, patients who reported moderate to severe sleep disturbance experienced a worse overall survival compared to those with no and mild sleep disturbance. The association was more pronounced in patients who were 50 years or older, male, overweight/obese, diagnosed with rectal cancer or stage I–III colorectal cancer. Notably, we did not observe significant associations between sleep disturbance and disease‐free survival and risk of recurrence, suggesting that sleep disruption may not directly influence tumor progression but could instead reflect broader systemic vulnerability or comorbid burden. Our study provides deeper insights into the limited and inconsistent literature on the relationship between sleep and mortality among colorectal cancer patients. To our knowledge, this is one of the largest and first population‐based studies to date to examine preoperative sleep disturbance and long‐term survival across all colorectal cancer stages, including patients undergoing curative‐intent surgery. These findings contribute novel evidence to a limited and inconsistent literature and highlight the potential prognostic relevance of patient‐reported sleep symptoms in pretreatment assessments.

Sleep disturbance affects approximately 60%–70% of colorectal cancer patients [12, 32], with rectal cancer patients reporting sleep complications more frequently than those with colon cancer [12]. However, to date, only a limited number of studies have explored the effect of sleep on clinical outcomes in colorectal cancer. Although this is one of the first studies to examine the role of sleep disturbance prior to surgery and survival among stage I–IV colorectal cancer survivors, one prior study investigated the impact of sleep disturbance among 361 chemo‐naïve patients with metastatic colorectal cancer [18]. In this post hoc analysis, sleep problems reported before the start of chemotherapy were associated with a higher risk of death (HR = 1.39; 95% CI = 1.11–1.74), disease progression (HR = 1.44; 95% CI = 1.16–1.78), and poor chemotherapy response (Odds Ratio (OR) = 0.58; 95% CI = 0.38–0.89) [18]. In contrast, our findings suggest a stronger association between sleep disturbance and overall survival in stage I–III patients, with no significant association observed in those with stage IV disease. This discrepancy may reflect differences in disease biology, prognosis, and treatment intent. Notably, while all participants in our study underwent surgery for the primary tumor, the rationale for surgical intervention in stage IV patients can vary widely, from curative resection of limited metastases to palliative procedures for management of symptoms. This heterogeneity likely influences survival outcomes and complicates interpretation in the metastatic subgroup. Additionally, the lack of statistically significant association in stage IV patients may also be due to reduced statistical power, given the smaller sample size. We have therefore highlighted this limitation and interpret our findings in stage IV patients with caution. Future research should further explore sleep‐related prognostic markers in more homogenous clinical populations or with detailed data on treatment intent.

Although studies on sleep disturbance and overall survival among colorectal cancer patients are limited, a few prior studies assessed the association of sleep duration with colorectal cancer outcomes, reporting inconsistent findings. In an analysis of 1175 stage III colon cancer patients enrolled in the CALGB/SWOG 80702 randomized adjuvant chemotherapy trial, very short (≤ 5 h) or very long (≥ 9 h) sleep duration was associated with worse overall survival HR = 2.14 (95% CI = 1.14–4.03) and HR = 2.34 (95% CI = 1.26–4.33), respectively [33]. Furthermore, a long sleep duration of ≥ 9 h was associated with shorter disease‐free survival (HR = 1.62; 95% CI = 1.01–2.58). On the other hand, no association between postdiagnosis sleep duration and overall survival or colorectal cancer‐specific mortality was found in a recent meta‐analysis, which included a range of cancer types [13]. In an analysis of self‐reported sleep duration and napping prior to diagnosis among 4869 colorectal cancer patients, a short sleep duration (< 5 h) was associated with a 36% increased risk in all‐cause mortality (HR = 1.36; 95% CI = 1.08–1.72) and a 54% risk in colorectal cancer‐specific mortality (HR = 1.54; 95% CI = 1.11–2.14) [19]. Further, daytime napping of > 1 h was associated with an increase in all‐cause mortality.

Sleep disturbances, particularly insomnia symptoms, have been linked to a higher risk of all‐cause mortality in the general population [34]. A large prospective study of 487,728 individuals from the UK Biobank found that frequent sleep disturbances were significantly linked to an increased risk of all‐cause mortality (HR = 1.13; 95% CI = 1.09–1.18) [34]. However, sleep disturbances often coexist with various comorbidities or contribute to the risk of developing conditions, which can independently increase mortality risk. For example, sleep disorders have been linked to cardiovascular disease (CVD), metabolic disorders, and immune dysfunction, which are significant contributors to overall mortality. A retrospective analysis of 138,201 individuals found that impaired sleep, defined as “difficulty falling asleep, staying asleep, or sleeping too much,” has been associated with an increased risk of obesity (OR = 1.18, *p* < 0.0005), diabetes (OR = 1.18, *p* < 0.005), coronary artery disease (OR = 1.59, *p* < 0.0005), stroke (OR = 1.22, *p* < 0.05), and myocardial infarction (OR = 1.36, *p* < 0.0005) [35], all of which can elevate the likelihood of noncancer‐related death. Furthermore, a study of 29,831 adults from the National Health and Nutrition Examination Survey found a strong association between sleep disturbances and an increased risk of CVD (OR = 1.74, 95% CI = 1.6 to 1.9; *p* < 0.001) [36]. In colorectal cancer patients, where multimorbidity is common [37], the association between sleep disturbances and overall survival may be linked not only to cancer‐related factors but also to other health conditions, particularly those affecting cardiometabolic health.

The underlying molecular mechanisms linking sleep disturbances to CRC survival remain unclear. However, previous studies suggest that sleep disturbances contribute to various physiological and psychological dysfunctions, which may play a role in disease progression. Chronic disruption of the circadian rhythm, often caused by irregular sleep patterns or insufficient sleep, impairs hormonal regulation, including cortisol and melatonin production [38], leading to dysregulation of the hypothalamic–pituitary–adrenal (HPA) axis [39]. This dysregulation heightens stress responses and promotes systemic inflammation, which is linked to cardiovascular disease, metabolic disorders [40], and cancer [41, 42]. These mechanisms may help explain the link between sleep disturbances and poorer overall survival in both cancer patients and the general population.

Our study presents several strengths alongside a few limitations. Key strengths include a large sample size, a multicenter design, a longitudinal approach, and the use of reliable, validated questionnaires to capture patient‐reported outcomes. Subjective sleep complaints were assessed using a specific item from a validated and widely used health‐related quality of life questionnaire, which is available in all relevant languages for the study population [25, 43]. The EORTC QLQ‐C30 includes a single self‐reported sleep question within a comprehensive, multidimensional patient‐rated questionnaire.

However, considering the diverse nature of sleep issues, relying on a single item, even though it is validated, may underestimate sleep disturbance compared to actigraphy or more comprehensive questionnaires and represents a limitation of this study. Future work should include objective measures of sleep disturbance, such as actigraphy or polysomnography. Another limitation is that, since this multicenter cohort study does not uniformly collect information regarding colorectal cancer specific or other causes of death, we were unable to explore the potential association between sleep disruption and colorectal cancer‐specific death or account for competing risks of death. Standardized operating procedures were used across all study centers to ensure consistent follow‐up of patients. These procedures included collecting clinical outcomes like overall survival through various sources such as medical chart reviews, death registries, or reports from external general practitioners. While efforts were made to account for key confounders, the potential for residual confounding cannot be entirely ruled out, as with other observational studies. For instance, underlying comorbidities linked to sleep disturbances may contribute to reverse causation. It is also important to note that while a differential association by age was observed, it may be due to limited sample size and not a true statistical interaction. Therefore, further investigation in larger, age‐diverse cohorts is warranted. Despite the acknowledged limitations, this study offers valuable practical implications for future research on colorectal cancer survivorship. In particular, behavioral interventions such as cognitive behavioral therapy for insomnia (CBT‐I) [44, 45], and exercise interventions [46, 47, 48] significantly alleviate sleep problems without the potential adverse effects of medication and could be promising management strategies for improving survival in colorectal cancer patients. Multiple studies and meta‐analysis have shown that physical activity is associated with improved survival outcomes among cancer patients [49, 50], including colorectal cancer patients [51].

In conclusion, moderate to severe sleep disturbance prior to surgery was associated with worse overall survival in colorectal cancer survivors, with stronger associations in those who were 50 years or older, male, overweight/obese, diagnosed with rectal cancer or stages colorectal I–III cancer. However, no significant association was observed with disease‐free survival or recurrence, suggesting that sleep disturbance may reflect broader health vulnerabilities or underlying comorbidities rather than directly influencing cancer progression. Given that only a single self‐reported item was used to assess sleep, and that comorbidities were not fully accounted for, our findings should be interpreted with caution. Rather than implying a causal effect, our results suggest that preoperative sleep disturbance may serve as a marker for identifying patients at higher risk of poor outcomes. Future studies are needed to validate these findings using more comprehensive sleep assessments and to determine whether screening for and addressing sleep disturbances in the preoperative setting can contribute meaningfully to risk stratification and survivorship care in colorectal cancer.

## Author Contributions

**Anita R. Peoples:** conceptualization (lead), data curation (lead), formal analysis (lead), investigation (lead), methodology (lead), software (lead), visualization (lead), writing – original draft (lead), writing – review and editing (lead). **Victoria Damerell:** conceptualization (lead), data curation (lead), formal analysis (lead), investigation (lead), methodology (lead), software (lead), writing – original draft (lead), writing – review and editing (lead). **Jennifer Ose:** formal analysis (equal), writing – review and editing (equal). **Erin M. Siegel:** funding acquisition (equal), writing – review and editing (equal). **Tengda Lin:** writing – review and editing (equal). **Sheetal Hardikar:** writing – review and editing (equal). **Caroline Himbert:** writing – review and editing (equal). **Mmadili N. Ilozumba:** writing – review and editing (equal). **Petra Schrotz‐King:** writing – review and editing (equal). **Sylvia L. Crowder:** writing – review and editing (equal). **Adetunji T. Toriola:** funding acquisition (equal), writing – review and editing (equal). **David Shibata:** funding acquisition (equal), writing – review and editing (equal). **Christopher I. Li:** funding acquisition (equal), writing – review and editing (equal). **Doratha A. Byrd:** funding acquisition (equal), writing – review and editing (equal). **Elena S. Aßmann:** writing – review and editing (equal). **Heather S. L. Jim:** writing – review and editing (equal). **Jane C. Figueiredo:** conceptualization (lead), funding acquisition (lead), project administration (lead), supervision (lead), writing – review and editing (equal). **Cornelia M. Ulrich:** conceptualization (lead), funding acquisition (lead), project administration (lead), supervision (lead), writing – review and editing (equal). **Biljana Gigic:** conceptualization (lead), funding acquisition (lead), project administration (lead), supervision (lead), writing – review and editing (equal).

## Funding

This study was supported by National Institutes of Health (NIH)/National Cancer Institute (NCI) grants (U01206110, R01 CA189184, R01 CA207371, R01 CA211705, T32 HG008962, KL2TR002539, K07 CA222060, R03AG067994), German Federal Ministry of Education and Research (BMBF) project PerMiCCion (01KD2101D), the Rahel‐Goitein‐Straus‐Program Medical Faculty Heidelberg University, Heidelberger Stiftung Chirurgie, Stiftung LebensBlicke, German Consortium of Translational Cancer Research (DKTK), German Cancer Research Center, Matthias Lackas Stiftung, Claussen‐Simon Stiftung, Huntsman Cancer Foundation, Immunology, Inflammation, and Infectious Disease Initiative at the University of Utah, ERA‐NET, JTC 2012 call on Translational Cancer Research (TRANSCAN). J. Ose received funding by NCI grant R03CA270473.

## Ethics Statement

The study was performed in accordance with the Declaration of Helsinki and approved by the institutional review board of the Moffitt Cancer Center (108437_MOD000047) and by the Ethics Committee of the University of Heidelberg (S‐134/2016), and all participants provided written informed consent. The ColoCare Study was registered on ClinicalTrials.gov Identifier: NCT02328677.

## Conflicts of Interest

Dr. Cornelia M. Ulrich has HCI Cancer Center Director oversight over research funded by several pharmaceutical companies but has not received funding directly herself. All other authors declare that they have no conflicts of interest.

## Data Availability

The ColoCare Study data is available from colocarestudy_admin@hci.utah.edu on reasonable request and as described on the ColoCare website (https://uofuhealth.utah.edu/huntsman/labs/colocare‐consortium/). Our data sharing procedures have been updated and are available online (https://uofuhealth.utah.edu/huntsman/labs/colocare‐consortium/data‐sharing/new‐projects.php). For any additional questions, please contact the ColoCare Study Administrator Team (colocarestudy_admin@hci.utah.edu).
